# Efficacy and safety of patiromer for hyperkalemia: a randomized, placebo-controlled phase 3 study

**DOI:** 10.1007/s10157-025-02637-4

**Published:** 2025-02-20

**Authors:** Naoki Kashihara, Hirokazu Okada, Yusuke Suzuki, Tamio Iwamoto, Masashi Yasutomi, Masaru Matsui, Ryo Takezawa, Takayuki Ishii, Yusuke Tomioka

**Affiliations:** 1 Department of Nephrology and Hypertension, Kawasaki Medical School, Okayama, Japan; 2 Faculty of Medicine, Department of Nephrology, Saitama Medical University, Saitama, Japan; 3 Department of Nephrology, Juntendo University School of Medicine, Tokyo, Japan; 4 Department of Nephrology and Hypertension, Saiseikai Yokohamashi Nanbu Hospital, Kanagawa, Japan; 5 Department of Nephrology, Kuwana City Medical Center, Mie, Japan; 6 Department of Nephrology, Nara Prefecture General Medical Center, Nara, Japan; 7 Zeria Pharmaceutical.Co., Ltd., 10–11, Nihonbashi Kobuna-cho, Chuo-ku, Tokyo, 103-8351 Japan

**Keywords:** Hyperkalemia, Patiromer, Phase III, Placebo-controlled

## Abstract

**Background:**

This is the phase 3 study in Japan designed to verify the superiority of patiromer over placebo using the change in serum potassium level (sK-level).

**Methods:**

This study was a multicenter, randomized withdrawal study targeting Japanese hyperkalemic patients. It consisted of the run-in period and the double-blind period. The run-in period was an active single-arm, open-label period (4 or 5 weeks). The double-blind period was a randomized, placebo-controlled, parallel-group, double-blind period (4 weeks). Patients whose sK-level was within the normal range at week 4 or 5 of the run-in period entered the double-blind period. Patients who entered the double-blind period were randomly assigned to the patiromer group or the placebo group.

**Results:**

As a result of the primary analysis, the change of the sK-level (95% CI) from baseline to week 4 in the double-blind-period, was – 0.02 (– 0.19, 0.15) mmol/L in the patiromer group, and 0.78 (0.60, 0.96) mmol/L in the placebo group, with a statistically significant difference between the two treatment groups (*p* < 0.001). Similarly, statistically significant differences were also observed between the two groups at weeks 1, 2, and 3. Furthermore, the proportion of patients whose sK-level was maintained within the normal range were statistically significantly higher in the patiromer group than in the placebo group at all time points. No adverse events requiring particular attention were observed.

**Conclusion:**

Patiromer can improve hyperkalemia by lowering sK-level and can suppress the recurrence of hyperkalemia with continued administration, and is safe and easy-to-use for a wide range of patients.

## Introduction

Hyperkalemia, a condition caused in part by chronic kidney disease (CKD), heart failure (HF), and renin–angiotensin–aldosterone system inhibitors (RAASi), is a serious electrolyte abnormality that can cause life-threatening arrhythmias, cardiac arrest, and sudden death [1, 2]. The higher the serum potassium level and the more advanced the stage of CKD, the higher the mortality rate. Furthermore, the use of RAASi is also recommended in CKD and HF guidelines to control disease progression and improve outcomes [3–6]. However, because RAASi contribute to hyperkalemia, more than 50% of hyperkalemic patients discontinue RAASi after their first hyperkalemic episode [7]. The limited use of conventional therapies for chronic hyperkalemia often forces reduction or discontinuation of RAASi in the management of hyperkalemia [8, 9].

Sodium polystyrene sulfonate (SPS) and calcium polystyrene sulfonate (CPS) have been commonly used potassium binders in Japan for over 50 years, but these products are poorly tolerated and their use can be associated with serious adverse reactions, including intestinal necrosis [10, 11]. Factors contributing to low tolerability include difficulty of intake, frequency of administration, and adverse events [7, 11].

In recent years, sodium zirconium cyclosilicate (SZC) has also been used as a new treatment for hyperkalemia, but it is suggested that sodium-containing medicines have been associated with cardiovascular risk [12]. Indeed, database studies suggested that sodium zirconium cyclosilicate might increase the risk of hospitalization for HF compared with patiromer in overseas [13, 14].

Patiromer is a non-absorbable, high-capacity potassium-binding polymer for oral suspension and is sodium-free because it is a drug that binds potassium in exchange for calcium. Patiromer is already approved in the United States, Europe, and several other countries for the treatment of hyperkalemia prior to Japan. As the patiromer is once daily dosing, it may overcome the issue of poor compliance in other potassium binders.

A phase 2 study in Japan confirmed that patiromer at a starting dose of 8.4 g was effective in lowering and maintaining serum potassium level (sK-level) within normal range for 1 year in Japanese hyperkalemia patients, including those on dialysis.

This is the phase 3 study in Japan designed to verify the superiority of patiromer over placebo using the change in sK-level as the primary endpoint.

## Materials and methods

### Study design

**Fig. 1:**
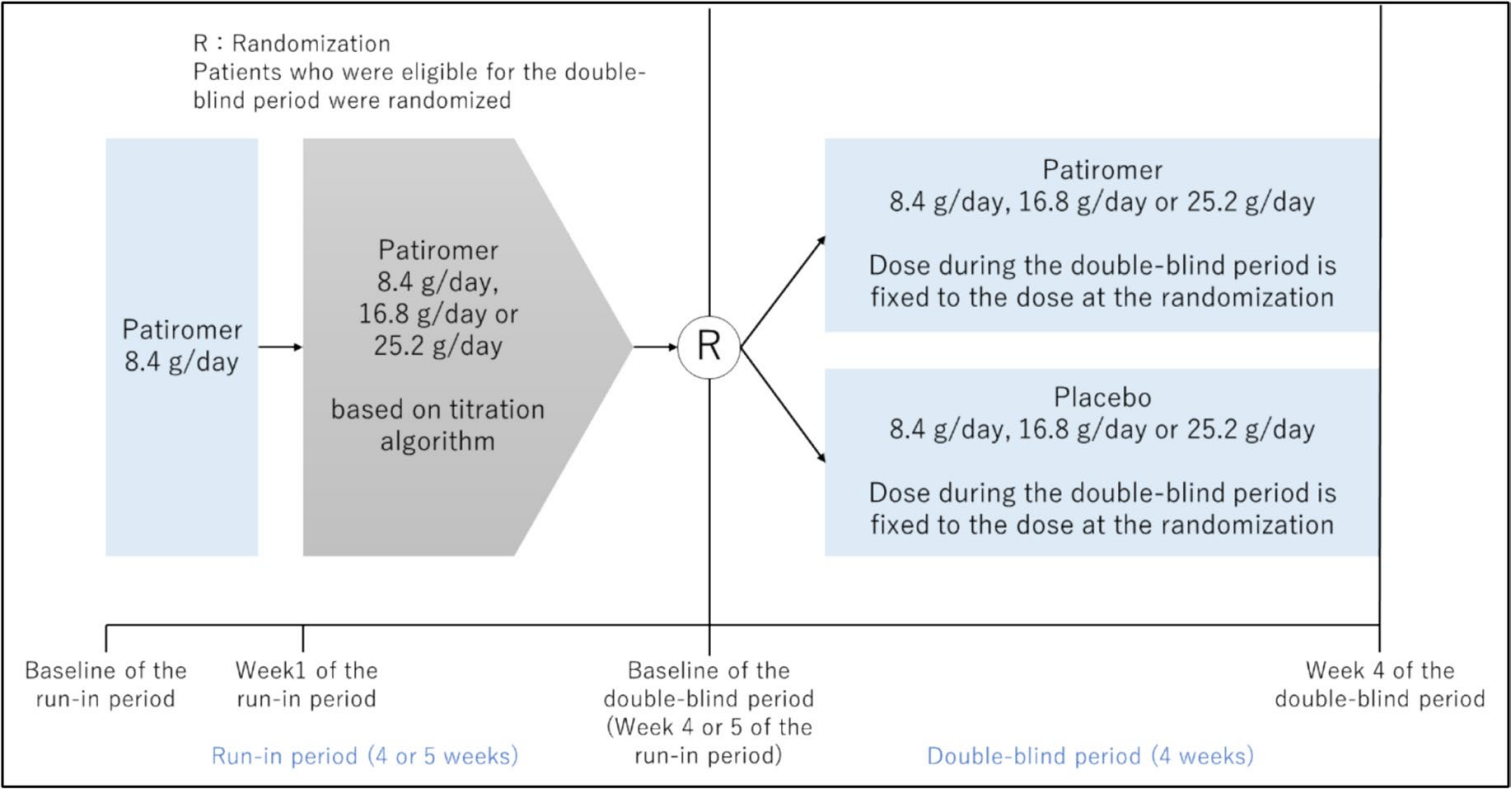
Study design

This study was a multicenter, randomized withdrawal study targeting Japanese hyperkalemic patients. It consisted of the run-in period and the double-blind period (Fig. 1). The run-in period is an active single-arm, open-label period. The double-blind period is a randomized, placebo (microcrystalline cellulose)-controlled, parallel-group, double-blind period.

This study was conducted in Japan between July 2021 and November 2022. This study is registered with ClinicalTrials.gov (NCT04955678).

The study protocol was approved by the local or central institutional review board for each participating site. All patients provided written informed consent; this study was performed in accordance with the ethical principles of the Declaration of Helsinki and current Good Clinical Practice and in compliance with local regulatory requirements.

### Patients

Patients aged 20 to 80 years with sK-level of 5.5 mmol/L or more but less than 6.5 mmol/L were eligible for this study.

Key exclusion criteria were anuria or history of acute renal insufficiency within 90 days prior to the run-in period baseline, severe gastrointestinal disorders, and use of SPS, CPS, SZC, and potassium supplement within 7 days prior to the run-in period baseline.

### Procedure, treatment, and randomization

Each samples was measured locally and centrally (both). Local measurements of sK-level were used for assessments of eligibility and dose adjustment. Central measurements of sK-level were used for assessments of efficacy and safety.

Patients who were confirmed to be eligible for this study entered the run-in period and began taking patiromer 8.4 g/day from baseline. During the run-in period, patients visited the investigator site once a week, and the patiromer dose was adjusted from 8.4 g/day to 25.2 g/day according to a predefined titration algorithm with sK-level (Supplement Table S1). Patients whose sK-level was within the normal range (3.5 or more and less than 5.1mmol/L) at week 4 or 5 of the run-in period entered the double-blind period.

Patients who entered the double-blind period were randomly assigned to the patiromer group or the placebo group (1:1) using dynamic allocation by the biased coin minimization method. The dose of the study drug during the double-blind period was fixed to the dose that each patient was taking at the end of the run-in period. The double-blind period was 4 weeks, with patients visiting the investigator site once a week. If the serum potassium level became less than 3.5, or 6.0 or more than during the double-blind period, administration of the study drug was discontinued at that point, considering the patient's safety.

The allocation factors were the baseline sK-level in the run-in period (“5.5 mmol/L or more but less than 6.0 mmol/L” / “6.0 mmol/L or more”), and the baseline sK-level in the double-blind period (“3.5 mmol/L or more and less than 4.3 mmol/L" / "4.3 mEq/L or more and less than 5.1 mEq/L").

The use of any treatment of hyperkalemia (except for the study drug) and potassium supplement was prohibited during the study period. Additionally, dose changes were prohibited for drugs that affect sK-level, such as RAASi, loop, thiazide and potassium sparing diuretics, non-selective beta-blockers, and SGLT2 inhibitors.

### Study endpoints

The primary efficacy endpoint was the change of sK-level from baseline to week 4 in the double-blind period. Other efficacy endpoints were the change in sK-level, the proportion of patients with sK-level within the normal range, and RAASi dose sustaining proportion. RAASi dose sustaining proportion was defined as proportion of patients who completed the double-blind period who did not reduce or discontinue RAASi and whose sK-level was less than 5.5 mmol/L throughout the double-blind period, among patients who were taking RAASi at the double-blind period baseline.

The safety endpoints were the incidence of adverse events (AEs) and adverse drug reactions (ADRs; AEs which were determined to be causally related to the study drug), blood tests (including serum magnesium, calcium and phosphorus), urine tests, vital signs, and resting 12-lead electrocardiogram.

### Statistical analysis

The statistical analysis plan was finalized prior to key break.

Efficacy analysis was performed on the full analysis set (FAS) (Supplementary Table S2).

The primary analysis was calculated by mixed effect models for repeated measures (MMRM), and *t* test using LSMean via MMRM between treatment groups was performed. For each treatment group, LSMean of the change of sK-level from baseline to week 4 in the double-blind period and its 95% CI of LSMean were calculated by MMRM. The model for the MMRM included the fixed, categorical effects of treatment, visit, and treatment-by-visit interaction, as well as the fixed, continuous covariates of baseline sK-level, with an unstructured covariance structure for the within-subject error.

The sample size needed for each treatment group in the double-blind period was estimated as 27 patients to perform the *t* test based on the following parameters: alpha = 0.05 (two-sided), power = 0.9, difference in changes from baseline to week 4 in the double-blind period in sK-level is 0.5, and standard deviation = 0.55. 30 per treatment group were derived considering to discontinuation. The parameters mentioned above refer to the data of the overseas phase 3 study [17]. Furthermore, the proportion of transition from run-in period to the double-blind period was estimated to be around 60–70%, so the target number of patients enrolled in the run-in period was 100.

Subgroup analyses by sK-level at the run-in period baseline and the double-blind period baseline, CKD stage, presence of diabetes mellitus, HF, hypertension and prior myocardial infarction, use of RAASi, and dose of the study drug were performed.

Safety analyses was performed on the safety analysis set (SAF) (Supplementary Table S2).

All analyses were conducted using SAS version 9.4 (SAS Institute Inc.).

## Results

A total of 173 patients were screened and 85 patients were determined eligible and entered the run-in period. Screening failure occurred in 88 patients, most of which was due to that patients had sK-level below 5.5 mmol/L. Of the 85 patients, 67 entered the double-blind period. Of the patients who did not enter the double-blind period, 5 patients discontinued administration of the study drug during the run-in period (Table 1). 13 patients did not meet the criteria for entering the double-blind period. Of the 13 patients, only one patient did not enter the double-blind period because the sK-level did not normalize. The remaining 12 patients did not enter the double-blind period because the sK-level (central) at the run-in period baseline did not meet the criteria (Table 2).

**Table 1:**
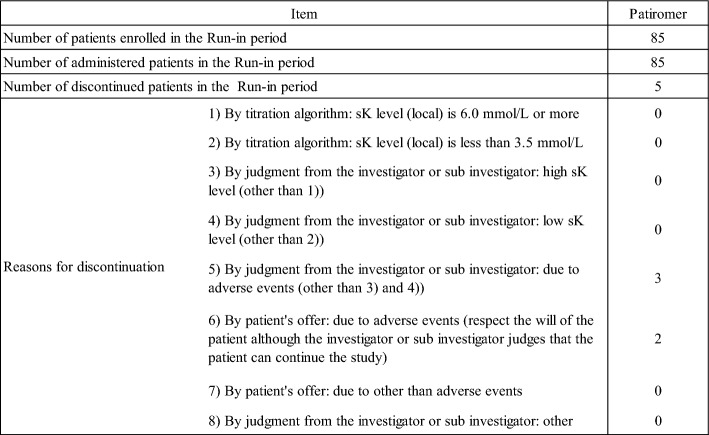
Reason for Discontinuation (Run-in period)

**Table 2:**
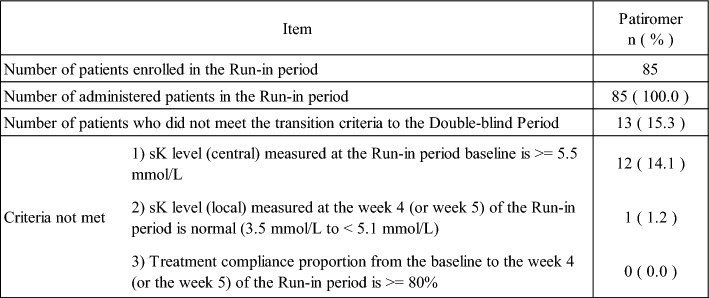
Reason for not entering to the double-blind period

(%): The denominator of the proportion is the number of patients enrolled in run-in period

**Fig. 2:**
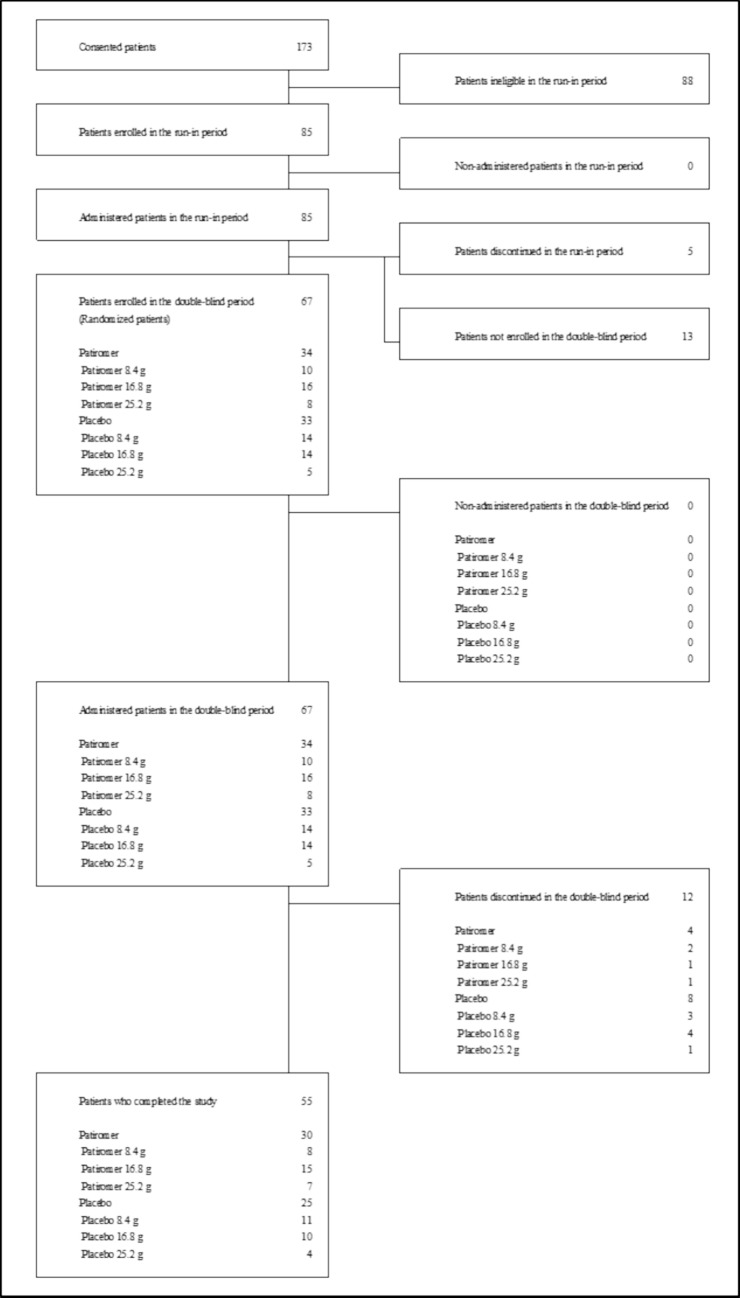
Disposition of patients

Of the 67 patients who entered the double-blind period, 34 were assigned to the patiromer group and 33 to the placebo group. 55 patients completed the study (30 in the patiromer group, 25 in the placebo group), and 12 patients discontinued the study. The reasons for discontinuing study drug administration during the double-blind period are shown in Table 3. The disposition of patients is shown in Fig. 2.

**Table 3:**
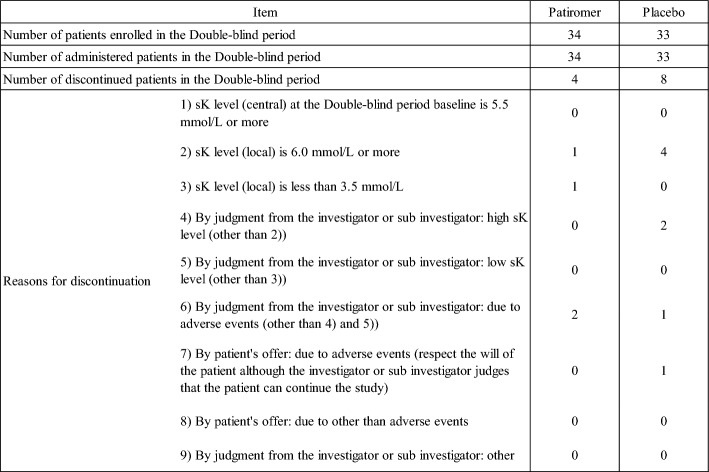
Reason for Discontinuation (Double-blind period)

The demographic and other baseline characteristics of the run-in period are shown in Table 4.

**Table 4:**
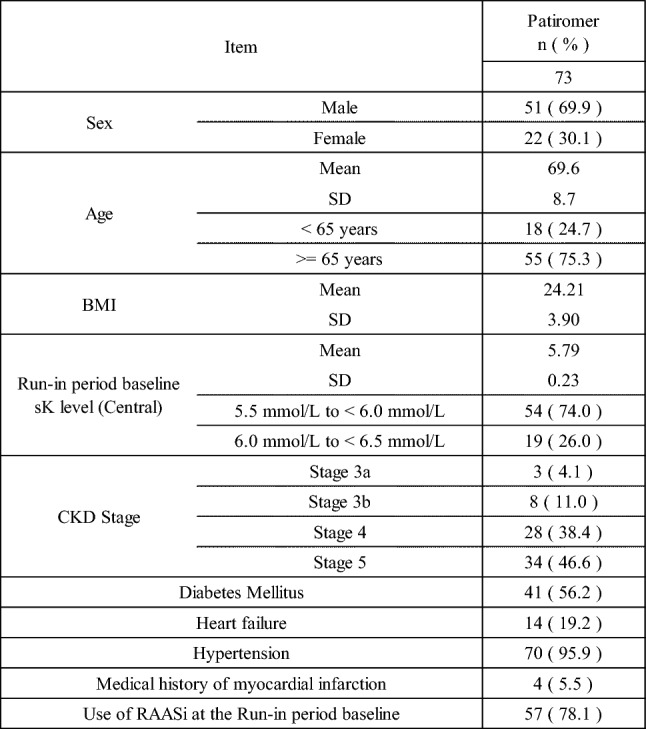
Demographic and Other Baseline Characteristics (Run-in period) (FAS)

The demographic and other baseline characteristics of the double-blind period were generally well balanced between the patiromer group and the placebo group (Table 5).

**Table 5:**
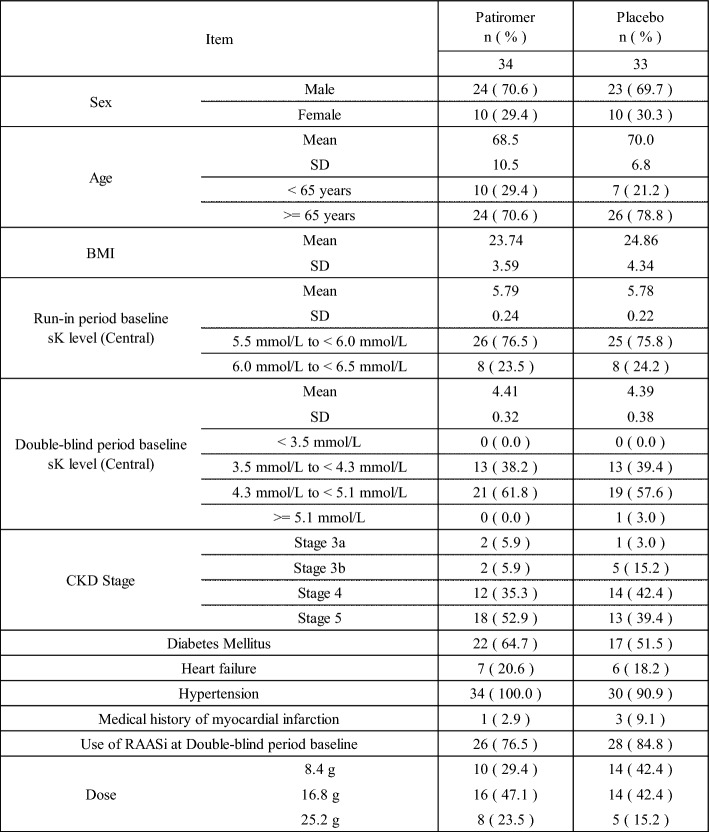
Demographic and Other Baseline Characteristics (Double-blind period) (FAS)

The mean compliance proportion was 99.43% during the run-in period, and 99.54% in the patiromer group, and 98.12% in the placebo group during the double-blind period (data not shown).

### Efficacy

#### Double-blind period

The double-blind period was a period in which patients whose sK-level has normalized during the run-in period were assigned to the patiromer group or the placebo group to evaluate efficacy.

As a result of the primary analysis, the change of sK-level LSMean (95% CI) from baseline to week 4 in the double-blind period was -0.02 (-0.19, 0.15) mmol/L in the patiromer group, and 0.78 (0.60, 0.96) mmol/L in the placebo group, with a statistically significant difference between the two treatment groups (*p* < 0.001) (Table 6).

**Table 6:**
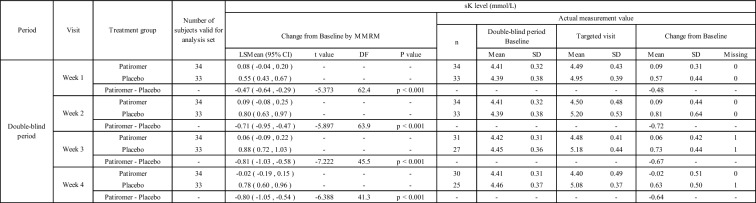
The Difference of the Change in sK-level for Each Visit of the double-blind period by MMRM

Change from baseline in sK = "sK-level at Targeted visit" – "baseline sK-level in the double-blind period."

*LSMean* Least Square Mean, *CI* Confidence Interval

Furthermore, regarding the change of sK-level LSMean from baseline to week 4 in the double-blind  period, the results for each subgroup had a similar trend to the results for the overall population (Figure S1).

The least squares mean change (95% CI) from baseline to week 1 of the double-blind period in sK values was 0.08 (-0.04, 0.20) mmol/L in the patiromer group and 0.55 (0.43, 0.67) mmol/L in the placebo group, demonstrating a statistically significant difference between the two groups at week 1. Similarly, statistically significant differences were observed between the two groups at weeks 2 and 3 (Table 6).

The proportion of patients whose sK-level was maintained within the normal range at week 1 of the double-blind period was 82.4% and 57.6%, and that at week 4 was 76.5% and 37.5%, respectively, in the patiromer group and in the placebo group. The proportion of patients whose sK-level was maintained within the normal range was statistically significantly higher in the patiromer group than in the placebo group at all time points, including weeks 2 and 3 (Table 7).

**Table 7:**
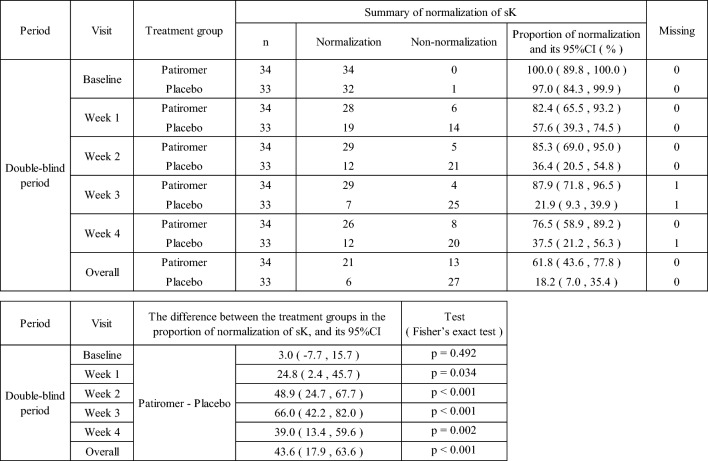
Maintenance of sK-level in the double-blind period (the normal sK: within 3.5 mmol/L to < 5.1 mmol/L, patients who discontinued are included in non-normalization)

The normal sK-level is defined as within 3.5 mmol/L to < 5.1 mmol/L

Overall: Throughout study of the double-blind period

For patients who discontinued the study, each sK-level until discontinuation is judged; however, after discontinuation, it is treated as non-normalization

The proportion (95% CI) of patients who were able to maintain the RAASi dose throughout the double-blind period was 84.6% (65.2, 95.6) and 53.6% (33.9, 72.4) in the patiromer group and the placebo group, respectively, and there was a statistically significant difference between the two treatment groups (*p* = 0.019) (Table 8).

**Table 8:**
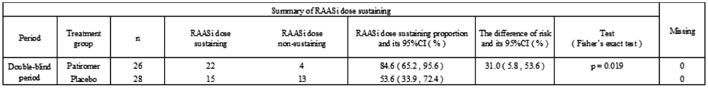
RAASi Dose Sustaining Proportion in the double-blind period

*n* the number of patients who were using RAASi at the double-blind period baseline

RAASi dose sustaining: The double-blind period completed patient in whom RAASi were not reduced or discontinued throughout the double-blind period and the sK-level was less than 5.5 mmol/L

RAASi dose non-sustaining: Throughout the double-blind period, patients who discontinued the study, or who reduced or discontinued RAASi, or whose sK-level breached over 5.5 mmol/L

RAASi dose sustaining proportion (%) = the number of "RAASi dose sustaining" / *n* × 100

**Fig. 3:**
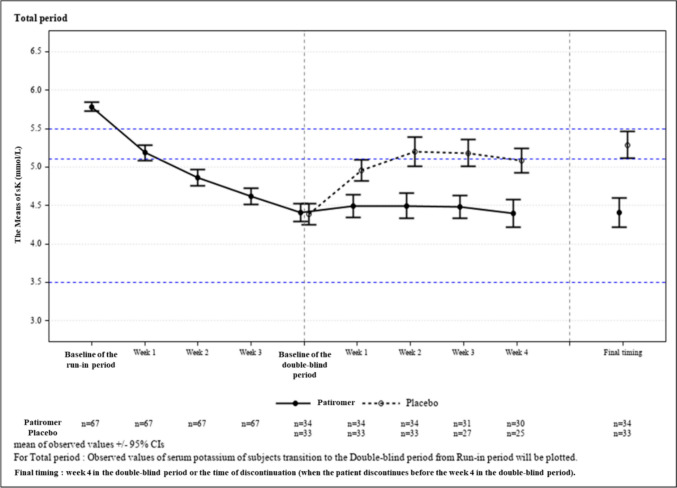
Means of sK (Total Period (From baseline of the run-in period to week 3 and from baseline to week 4 in the double-blind period, and Final Timing))

Figure 3 shows the change in mean sK-level during the study period (including run-in period) for patients who entered the double-blind period.

#### Run-in period

The run-in period was a period in which all patients were administered the patiromer.

The change in serum potassium level from baseline during the run-in period was – 0.61 mmol/L at week 1, – 0.94 mmol/L at week 2, and – 1.16 mmol/L at week 3, – 1.29 mmol/L at week 4, and – 1.35 mmol/L at week 5 of the run-in period. Because most patients entered the double-blind period at week 4 of the run-in period, the number of patients at week 5 was small compared to other weeks (Table 9).

**Table 9:**
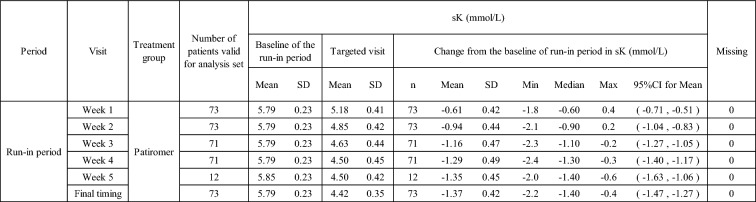
Change in sK-level in run-in period by Time (Weeks 1 to 5, and Final Timing of the run-in period)

Mean and SD of "the baseline of the run-in period" and "Targeted Visit," and Change from baseline in sK: Patients whose values for both "the baseline of the run-in period" and "Targeted Visit" were not missing were analyzed. Patients whose level(s) were missing were shown to the "Missing."

Change from baseline in sK = "sK-level at Targeted visit" – "sK-level at the baseline of the run-in period."

Final timing: The week 4 (or the week 5 if any) of the run-in period or the time of discontinuation (when the patient discontinues before the week 5 of the run-in period)

The proportion of patients whose sK-level became normal was 32.9% at week 1, 74.0% at week 2, 83.1% at week 3, 85.9% at week 4, and 91.7% at week 5 of the run-in period. Furthermore, the proportion of patients whose sK-level became normal at the final timing of each patient during the run-in period was 97.3% (Table 10).

**Table 10:**
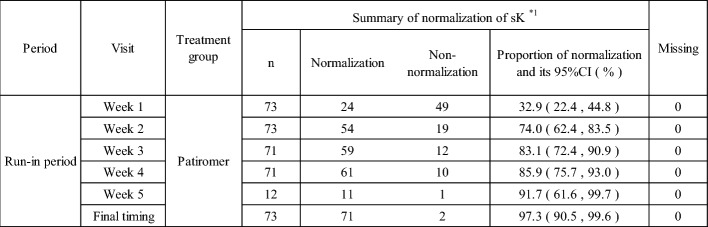
Normalization of Serum Potassium in the run-in period (the Normal Serum Potassium: within 3.5 mEq/L to < 5.1 mEq/L)

Final timing: The week 4 (or the week 5 if any) of the run-in period or the time of discontinuation (when the patient discontinues before the week 5 of the run-in period)

The normal sK-level is defined as within 3.5 mmol/L to < 5.1 mmol/L

Patients who transit to the double-blind period from the run-in period week 4 are not included in the week 5 of the run-in period

*1: For patients who discontinued the study, each sK-level until discontinuation is judged; however, after discontinuation, it is excluded from the analysis

Furthermore, the management target for sK-level is recommended to less than 5.5 mmol/L [15], and in this study, the proportion of patients whose sK-level was 3.5 or more and less than 5.5 mmol/L during the run-in period was 74.0% at week 1, 93.2% at week 2, 97.2% at week 3, 95.8% at week 4, and 100.0% at week 5 of the run-in period (data not shown).

### Safety

Safety was evaluated in 85 patients of SAF.

During the run-in period, the incidence of AEs was 32.9%, the incidence of ADRs was 12.9%, the incidence of serious AEs was 2.4%, and the incidence of serious ADRs was 1.2%.

During the double-blind period, the incidence of AEs was 32.4% and 24.2%, of ADRs was 8.8% and 0.0%, and of serious AEs was 5.9% and 0.0% in the patiromer group and the placebo group, respectively. No serious ADRs were observed in any group (Table 11).

**Table 11:**
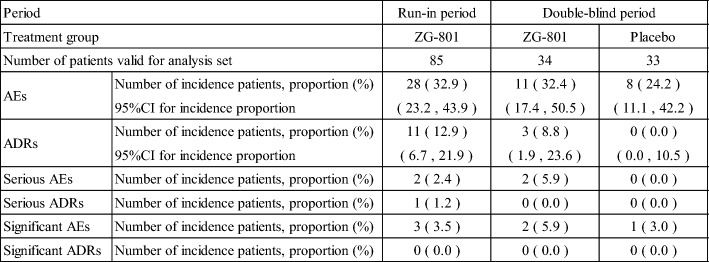
Brief Summary of Adverse Events

Number of incidence patients: If one patient with two or more same events, it was counted only once

Incidence proportion (%) = Number of incidence patients / Number of patients valid for analysis set × 100

ADRs: AEs that have been determined to be “related” to the study drug

Significant AEs: AEs leading to discontinuation of administration, including serious AEs

Significant ADRs: ADRs leading to discontinuation of administration, including serious ADRs

No deaths occurred in this study. Furthermore, there were no serious AEs that occurred in multiple patients, and serious ADR was only "decreased appetite (1/85 patients)" in the run-in period.

The ADR that occurred in 5% or more during the total study period was only "constipation (10.6%, 9/85 patients)," none of which were serious, and the severity was mild in 8/9 patients and was moderate in 1/9 patients (Table 12).

**Table 12:**
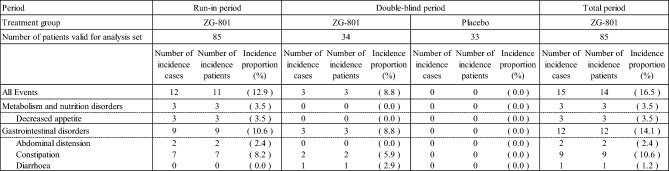
ADRs of Each Preferred Term

Event name: MedDRA Ver.24.0

Number of incidence cases: If one patient with two or more same events, it was all counted

Number of incidence patients: If one patient with two or more same events, it was counted only once

Incidence proportion (%) = Number of incidence patients / Number of patients valid for analysis set × 100

Total period: excluding data from the placebo group during the double-blind period

In this study, as AEs, HF and peripheral edema were observed in 1.2% (1/85 patients), and edema was observed in 2.4% (2/85 patients) during the study period, but none of the events were determined to be causally related to the study drug (data not shown).

Only 2.4% (2/85 patients) of the patients had sK-level below 3.5 mmol/L at least once during the study period, and no patients had sK-level below 3.0 mmol/L (data not shown). Furthermore, no symptoms related to low sK-level were observed in any of the patients.

Hypomagnesemia was not reported as adverse event in this study. Other blood tests including serum calcium and phosphorus, urine tests, vital signs, and resting 12-lead electrocardiogram showed no notable safety risks associated with administration of the study drug.

## Discussion

The primary endpoint, the change of sK-level from baseline to the week 4 in the double-blind period, was significantly higher in the placebo group compared to the patiromer group. Therefore, it was verified that continuing patiromer administration can significantly suppress the rise in sK-level compared to discontinuing patiromer administration and switching to placebo administration.

At all visits during the double-blind period, increases in sK-level from the double-blind period baseline were significantly lower in the patiromer group compared to the placebo group. Additionally, at all visits during the double-blind period, the proportion of patients with sK-level within the normal range was significantly higher in the patiromer group than in the placebo group. Therefore, by continuing patiromer administration, the sK-level can be maintained within the normal range. The change in sK-level from baseline to the week 1 and the proportion of patients whose sK-level was maintained within the normal range at the week 1 in the placebo group suggested that sK-level could rise early when patiromer was interrupted, so it is suggested that it is important to continue administering patiromer without interruption as much as possible.

Considering the results of the run-inperiod, it was suggested that patiromer was able to reduce sK-level to less than 5.5 mmol/L, the management target recommended by the guidelines, in most patients within one week. And it was suggested that patiromer was able to reduce sK-level to within the normal range in most patients within two weeks by adjusting the dose of patiromer as needed.

Furthermore, the proportion of patients who were able to maintain the RAASi dose throughout the double-blind period was higher in the patiromer group compared to the placebo group. In this study, changes in the dose of RAASi were prohibited during the study period in order to exclude factors that could affect the change in sK-level, which was the primary endpoint. Therefore, RAASi dose reduction or discontinuation did not generally occur, and the RAASi dose sustaining effect of the patiromer was difficult to accurately evaluate by only evaluating the proportion of "RAASi dose reduction patients" and "RAASi discontinuation patients." Therefore, we assumed that patients whose sK-level reached 5.5 mmol/L or higher were patients who needed to reduce or discontinue RAASi, and evaluated the RAASi dose sustaining proportion. This is based on the fact that reducing or discontinuing RAASi is recommended as one of the therapeutic options when sK-level reaches 5.5 mmol/L or higher while using RAASi [15]. Considering the above, it had been suggested that controlling sK-level with patiromer could enable continuous administration of RAASi, so patiromer could be a useful drug clinically for hyperkalemic patients with CKD and chronic HF who require RAASi.

Regarding safety, overall, no AEs requiring particular attention were observed, suggesting that it is a highly safe drug. The patiromer dosing regimen starts at the lowest dose and is increased appropriately based on each patient's sK-level, which might help minimize the occurrence of side effects.

Serious gastrointestinal disorders such as intestinal perforation, which are a problem with existing hyperkalemia treatments CPS and SPS, were not observed in this study.

Although the ADR that occurred most frequently was constipation, it was to be sufficiently controllable by using laxatives as needed, because no serious events were observed, the severity of constipation was mild in most patients, and no patients discontinued administration of the study drug due to constipation.

Hypokalemia is an important ADR related to life prognosis. Only 2.4% of patients had serum potassium levels below 3.5 mmol/L at least once during the study period, no patients had serum potassium levels below 3.0 mmol/L, and no symptoms related to low sK-level were observed in any of the patients. Therefore, the risk of developing hypokalemia due to administration of patiromer is low, and it was also confirmed that even if hypokalemia occurs, it can be managed by adjusting the dose of patiromer as necessary.

Additionally, ADRs such as congestive HF, hypertension, and edema caused by sodium ions, which are problems with sodium-containing products such as SPS and SZC, were not observed in this study. Therefore, patiromer can be an easy-to-use drug for patients with CKD who also have HF, hypertension, edema, etc.

Patiromer showed sufficient efficacy against hyperkalemia by administration of only once daily, without any initial loading dose adjustment, and had no significant safety risks, making it an easy-to-use treatment option that is expected to improve compliance compared to existing hyperkalemia drugs.

A database study showed that medical costs for patients with hyperkalemia increased over the long term, and the frequency of recurrent hyperkalemia contributed to the increase in medical costs, so it was important to continuously manage sK-level within the normal range [15]. Another Japanese database study found that old potassium binders had poor compliance and were not administered at the required dose for hyperkalemia treatment [7]. Patiromer is expected to be high compliance because of administration once daily and is a safe drug, which may enable long-term administration, thereby preventing the recurrence of hyperkalemia and might making it an excellent drug from a medical economic perspective.

This study has several limitations. Dietary potassium intake and urinary potassium excretion were not evaluated; however, patients were instructed not to change eating habits so far before and after participating in this study every visit. The initiation, dose change, and discontinuation of diuretics and NSAIDs were prohibited during the study. There were no specific restrictions on defecation control.

## Conclusion

Patiromer is a drug that can improve hyperkalemia by lowering sK-level and can suppress the recurrence of hyperkalemia with continued administration. Patiromer has been suggested to be an effective, safe, no sodium load, and easy-to-use drug for long-term treatment to a wide range of patient populations, especially hyperkalemia patients with CKD and HF.
